# Characterizing MicroPure ultrasound detectability of mineralization-related echogenic foci in regenerate bone during tibial bone transport

**DOI:** 10.3389/fmed.2026.1916995

**Published:** 2026-08-26

**Authors:** Ze Zhang, Juan Li, Dong Wang, Hongjie Zhou, Yonghong Zhang

**Affiliations:** 1 Second Clinical Medical College, Shanxi Medical University, Taiyuan, Shanxi, China; 2 Department of Orthopaedics, Second Hospital of Shanxi Medical University, Taiyuan, Shanxi, China; 3 Department of Ultrasound, The Second Hospital of Shanxi Medical University, Taiyuan, China

**Keywords:** distraction osteogenesis, healing index, MicroPure ultrasound, mineralization-related echogenic foci, musculoskeletal ultrasound, regenerate bone, tibial bone transport

## Abstract

**Background:**

Early assessment of regenerate bone mineralization is essential for monitoring distraction osteogenesis and guiding clinical management during tibial bone transport. MicroPure ultrasound is an advanced imaging technique designed to enhance the visualization of microcalcifications in soft tissues; however, its detection characteristics and clinical value in regenerate bone remain unclear. This study aimed to explore the size and depth-related factors associated with MicroPure detectability and identify exploratory classification thresholds associated with MicroPure detectability of early mineralization-related echogenic foci in regenerate bone, and investigate their association with healing outcomes.

**Methods:**

A retrospective study was conducted in 15 patients (16 tibiae) undergoing tibial bone transport for chronic osteomyelitis. Thirty-two ultrasound examinations were performed at 2 and 4 weeks after distraction initiation. A total of 1,976 mineralization-related echogenic foci identified on conventional B-mode ultrasound were analyzed, including 988 MicroPure-detectable and 988 MicroPure-undetectable echogenic foci. Maximum diameter and depth were compared between groups. Receiver operating characteristic (ROC) analysis was used to determine exploratory classification thresholds, and generalized estimating equations (GEE) were performed to evaluate factors independently associated with MicroPure detectability. Associations between early MicroPure-detectable MREF counts and healing index were evaluated using linear regression.

**Results:**

MicroPure-detectable echogenic foci exhibited significantly smaller diameters and shallower depths than MicroPure-undetectable echogenic foci (both *p* < 0.001). ROC analysis identified exploratory classification thresholds of 1.25 mm for diameter (AUC = 0.886) and 0.925 cm for depth (AUC = 0.756). Both diameter (OR = 0.002, 95% CI 0.001–0.005) and depth (OR = 0.089, 95% CI 0.016–0.492) were independently associated with MicroPure detectability. At the tibia level, greater numbers of early MicroPure-detectable echogenic foci were significantly associated with lower healing indices in both transverse (R^2^ = 0.511, *p* = 0.002) and longitudinal scans (R^2^ = 0.380, *p* = 0.011).

**Conclusion:**

MicroPure ultrasound showed greater detectability for smaller and more superficial mineralization-related echogenic foci in regenerate bone. In this cohort, exploratory classification thresholds of 1.25 mm for diameter and 0.925 cm for depth were identified, providing quantitative reference values for interpreting MicroPure findings during tibial bone transport. Furthermore, a greater burden of early MicroPure-detectable echogenic foci was associated with more favorable healing outcomes. These findings support the potential role of MicroPure ultrasound as a non-invasive tool for early mineralization assessment and monitoring during distraction osteogenesis.

## 1 Introduction

Bone transport based on the principles of distraction osteogenesis is an established technique for the reconstruction of segmental bone defects resulting from trauma, chronic osteomyelitis, tumor resection, and other complex orthopedic conditions (1–3). By gradually distracting an osteotomized bone segment using an external fixator, new bone is generated within the distraction gap, ultimately restoring skeletal continuity and limb function (4, 5). Despite its effectiveness, the success of bone transport largely depends on the quality and rate of regenerate bone formation. Delayed mineralization and poor regenerate consolidation remain major causes of prolonged external fixation, treatment failure, and increased complication rates (6). Therefore, reliable methods for early assessment of regenerate bone mineralization are of considerable clinical importance. Conventional radiography is the most widely used imaging modality for monitoring distraction osteogenesis. However, radiographic evidence of mineralization generally becomes apparent only several weeks after distraction begins, limiting its ability to detect early biological changes within the regenerate bone (7, 8). Moreover, radiographs primarily provide morphological information and are relatively insensitive to microscopic mineral deposition during the initial stages of bone formation. Computed tomography (CT) and dual-energy X-ray absorptiometry (DEXA) may offer more quantitative assessments of mineralization, but their routine use is constrained by radiation exposure, cost, and limited accessibility. Consequently, there remains a need for non-invasive techniques capable of detecting early mineralization activity before conventional radiographic signs become evident. Ultrasound has emerged as a promising tool for monitoring bone regeneration because of its real-time capability, lack of ionizing radiation, portability, and repeatability (9, 10). Previous studies have demonstrated that ultrasound can visualize early callus formation, periosteal reactions, and vascular changes during fracture healing and distraction osteogenesis. Nevertheless, conventional B-mode ultrasound has limited sensitivity for identifying minute mineral deposits, particularly during the earliest phases of regenerate bone formation when these changes remain sparse and heterogeneous.

MicroPure ultrasound imaging is an advanced image-processing technology designed to enhance the visualization of microcalcifications. By applying dedicated image-processing algorithms, MicroPure enhances the visualization of small calcification-related echoes against surrounding soft tissues (11–13). The technique has been extensively investigated in breast and thyroid imaging, where it has demonstrated improved detection of microcalcifications compared with conventional ultrasound (14–16). However, the performance of MicroPure in the unique acoustic environment of distraction osteogenesis has not been evaluated. In particular, the size- and depth-related factors influencing MicroPure detectability in regenerate bone remain unclear. Establishing quantitative classification thresholds associated with MicroPure detectability may help clinicians better interpret MicroPure findings and recognize the technical characteristics of this imaging modality. Unlike previous applications of MicroPure ultrasound that have primarily focused on superficial organs such as the breast and thyroid, the present study represents the first investigation, to our knowledge, of MicroPure imaging characteristics in regenerate bone during tibial bone transport. Unlike soft-tissue microcalcification assessment, imaging of distraction regenerate presents unique challenges because early mineralization occurs within a mechanically active and continuously changing bone environment. Therefore, understanding the technical factors influencing MicroPure detectability is important for evaluating the potential application of this technology in monitoring distraction osteogenesis. By analyzing paired B-mode and MicroPure ultrasound images, this study sought to characterize the imaging factors associated with MicroPure detectability and to explore the potential clinical relevance of early MicroPure-detectable mineralization-related echogenic foci (MREFs) in relation to regenerate consolidation. Therefore, the primary objective of this study was to investigate the size- and depth-related factors associated with MicroPure detectability and to identify exploratory classification thresholds for early MREFs during tibial bone transport. The secondary objective was to explore the association between early MicroPure-detectable MREF burden and healing outcomes, as measured by the healing index. Through these analyses, we sought to provide an initial quantitative framework for interpreting MicroPure images in distraction osteogenesis and to establish a foundation for future prospective validation studies.

## 2 Materials and methods

### 2.1 Study design and participants

This retrospective observational study was conducted in accordance with the Strengthening the Reporting of Observational Studies in Epidemiology (STROBE) statement. The study protocol was approved by the Institutional Ethics Committee of the Second Hospital of Shanxi Medical University (Approval No. 2024YX351). The requirement for written informed consent was waived according to the approved research protocol because this study involved retrospective analysis of existing clinical and imaging data. The approved protocol included retrospective analysis of clinical, imaging, and follow-up data from patients who underwent tibial bone transport between March 2023 and September 2024.

Patients who underwent tibial bone transport for chronic osteomyelitis under the care of the same orthopedic team between March 2023 and September 2024 were identified from the institutional database and assessed for eligibility according to predefined inclusion and exclusion criteria. Because MicroPure examinations were introduced only in this clinical team during the study period, inclusion was based on the availability of paired B-mode and MicroPure ultrasound examinations rather than a broader screening cohort. During this period, MicroPure ultrasound examinations were performed only in patients treated by this team; therefore, only patients with available paired B-mode and MicroPure ultrasound examinations were considered eligible for the present analysis. Eligible patients met the following criteria: (1) age between 18 and 80 years; (2) tibial bone defect length ≥ 3 cm; and (3) treatment with bone transport using a circular external fixator. Exclusion criteria were: (1) inability to comply with treatment or follow-up; (2) severe medical conditions precluding surgical treatment; (3) inadequate soft tissue conditions preventing ultrasound examination; and (4) incomplete clinical or imaging data.

**FIGURE 1:**
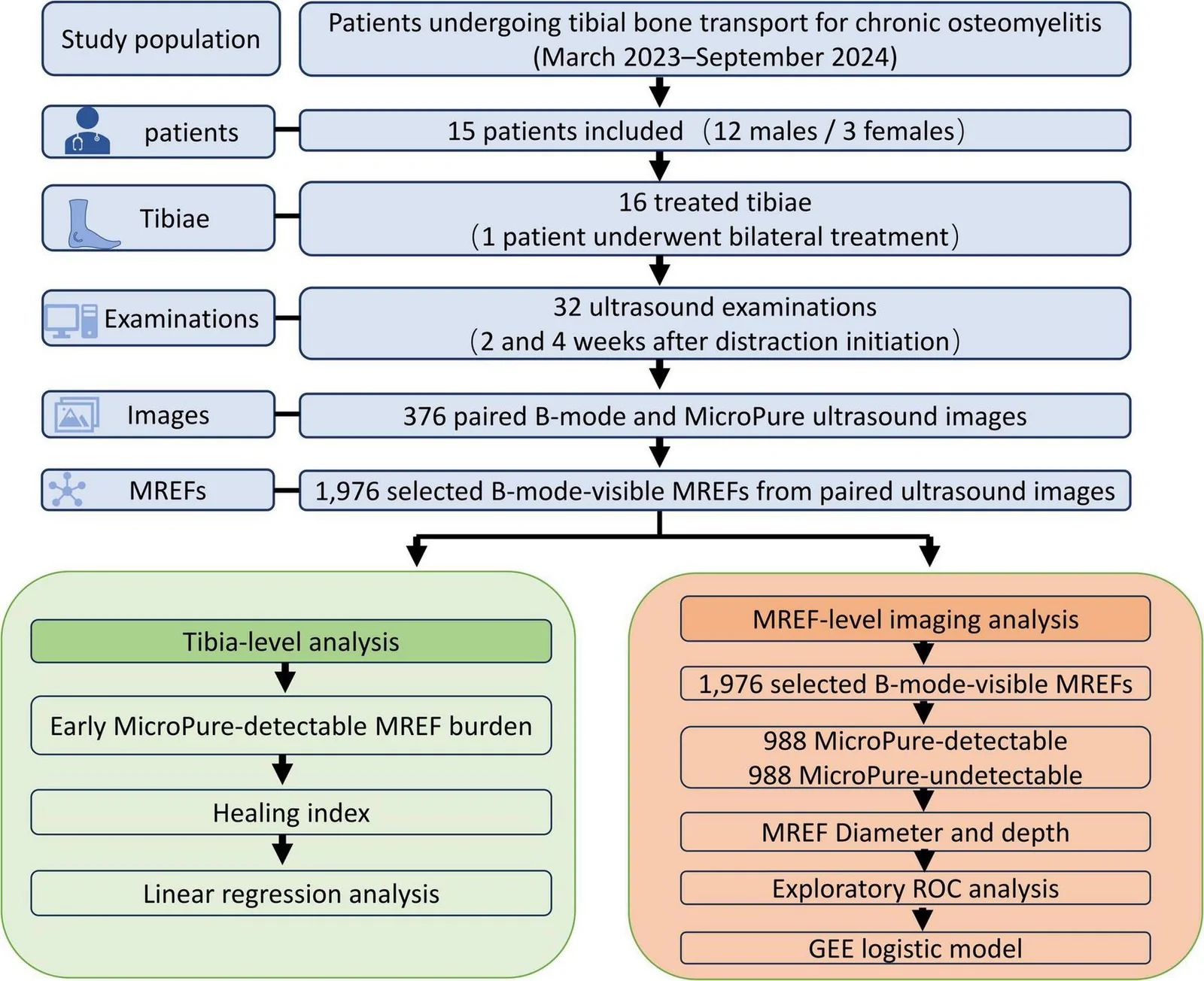
Study design and analytical workflow. Fifteen patients undergoing tibial bone transport for chronic osteomyelitis between March 2023 and September 2024 were included in this retrospective study. A total of 16 tibiae and 32 ultrasound examinations performed at 2 and 4 weeks after distraction were analyzed. B-mode ultrasound and MicroPure imaging were used to evaluate regenerate bone. Two levels of analysis were conducted. Tibia-level analysis assessed the association between early MicroPure-detectable MREF counts and healing index. Foci-level analysis included 1,976 MREFs (988 MicroPure-detectable and 988 MicroPure-undetectable MREFs) and evaluated the associations between MREF diameter, depth, and MicroPure detectability.

A total of 15 patients were included. One patient underwent bilateral treatment, resulting in 16 tibiae available for analysis (Figure 1). Demographic and clinical characteristics, including age, sex, bone defect length, comorbidities, and smoking history, were collected from medical records.

### 2.2 Bone transport procedure

All surgical procedures were performed by the same orthopedic team using circular external fixation systems. Following radical debridement and osteotomy, bone transport was initiated according to standard distraction osteogenesis protocols. Distraction began on postoperative day 12 and proceeded at a rate of approximately 0.83 mm/day, divided into five adjustments per day. Bone transport continued until docking site closure and restoration of bone continuity. External fixator removal was performed after confirmation of regenerate consolidation and docking-site union based on radiographic evaluation and clinical assessment by the treating surgeons.

### 2.3 Ultrasound examination

Ultrasound examinations were performed using an Aplio 500 ultrasound system (Canon Medical Systems, Japan) equipped with a PLT-704SBT linear transducer operating at 7.5–13 MHz. The ultrasound frequency, focal position, imaging depth, and overall gain were adjusted according to patient anatomy and image quality during routine clinical examinations. MicroPure-specific parameters, including sensitivity, detection threshold, blending parameters, and gain, were maintained at the manufacturer’s default settings throughout the study. The same ultrasound system and transducer were used for all examinations. Imaging depth and focal position were adjusted according to patient anatomy and image quality to optimize visualization of the regenerate zone. Conventional B-mode and MicroPure images were acquired simultaneously during each examination. Each patient underwent ultrasound evaluation at 2 and 4 weeks after initiation of distraction. During examination, patients were placed in the supine position with the affected limb fully exposed. The regenerate bone zone was scanned in both longitudinal and transverse planes. In the present study, B-mode ultrasound-visible hyperechoic foci were termed mineralization-related echogenic foci (MREFs), which were considered potential imaging markers of early regenerate bone mineralization rather than confirmed histological mineral deposits. All examinations were performed by two experienced musculoskeletal sonographers. The presence or absence of MicroPure detectability was assessed through review of paired B-mode and MicroPure images by two experienced musculoskeletal sonographers. Disagreements were resolved by consensus. The maximum diameter and depth of each MREF were measured by one investigator using electronic calipers on B-mode ultrasound images. Measurements were repeated three times, and the average value was used for subsequent analyses.

### 2.4 Selection of MREFs for foci-level analysis

The objective of this analysis was to explore the size- and depth-related factors associated with MicroPure detectability and to identify exploratory classification thresholds within B-mode ultrasound-visible MREFs. A total of 376 paired B-mode and MicroPure ultrasound images from 32 examinations were reviewed. Because individual MREFs could not be reliably spatially matched between the 2-week and 4-week examinations, longitudinal tracking of individual foci was not performed. Therefore, the foci-level analysis was intended to characterize factors associated with MicroPure detectability rather than longitudinal changes in individual MREFs. For each eligible image, up to three MicroPure-detectable MREFs and three MREFs visible on B-mode ultrasound but not detected by MicroPure were selected for comparative analysis. When fewer than three eligible MREFs were available in either category, all available MREFs were included. This balanced sampling strategy was designed to compare imaging characteristics associated with MicroPure detectability rather than estimate the prevalence of detectable or undetectable foci. Finally, a total of 1,976 MREFs were included, comprising 988 MicroPure-detectable and 988 MicroPure-undetectable MREFs. The hierarchical structure of the dataset was defined as follows: patients represented the clinical unit, tibiae represented the treatment unit, examinations and ultrasound images represented imaging acquisition units, and MREFs represented imaging-level observations.

#### 2.4.1 Measurement of MREF diameter

The maximum diameter of each MREF was measured directly on stored ultrasound images using the electronic caliper integrated into the ultrasound system. Measurements were performed by a single investigator on the image plane where the MREF appeared most clearly. Values were recorded to the nearest 0.1 mm, and three repeated measurements were obtained, with the average value used for subsequent analyses.

#### 2.4.2 Measurement of MREF depth

MREF depth was defined as the perpendicular distance from the skin surface to the center of the MREF. Because exported ultrasound images were available only in BMP format without embedded DICOM spatial information, depth measurements were performed using ImageJ software (National Institutes of Health, Bethesda, MD, USA). The scale was calibrated using the 1-cm reference marker displayed on each image. Following calibration, the distance from the skin surface to the center of each MREF was measured by a single investigator.

#### 2.4.3 Classification of MREF detectability

MREFs were classified into two groups:

1. MicroPure-detectable MREFs (visible on MicroPure imaging);
2. MicroPure-undetectable MREFs (visible on conventional B-mode ultrasound but not on MicroPure imaging).

### 2.5 Tibia-level analysis

The secondary objective of the study was to explore the association between early MicroPure-detectable MREF burden and healing outcomes. Because each transported tibia represented an individual treatment course, healing-related analyses were performed at the tibia level. One patient contributed bilateral tibiae, and each treated tibia was considered as a separate treatment unit. For each tibia, the number of MicroPure-detectable MREFs was recorded separately in longitudinal and transverse scans. Ultrasound examinations were performed using a consistent clinical scanning approach, with the regenerate zone systematically evaluated in both longitudinal and transverse planes. However, because the retrospective dataset did not include predefined quantitative acquisition parameters such as scanned length, scanned area, or regenerate volume, MREF counts were interpreted as relative indicators of MicroPure-detectable echogenic activity rather than absolute measures of mineralization density. Individual MREFs were not longitudinally tracked between the 2-week and 4-week examinations because reliable spatial correspondence could not be established retrospectively. Therefore, measurements obtained at 2 and 4 weeks were averaged to represent the early MREF burden.

Healing outcome was assessed using the healing index, calculated as: Healing Index = Total duration of external fixation (days)/Bone defect length (cm).

Associations between MREF counts and healing index were evaluated separately for longitudinal and transverse scans. Given the limited sample size, these analyses were considered exploratory, and multivariable adjustment for potential clinical confounders was not performed.

### 2.6 Statistical analysis

Statistical analyses were performed using SPSS version 27.0 (IBM Corp., Armonk, NY, USA).

For MREF-level analyses, continuous variables were expressed as mean ± standard deviation or median (interquartile range), depending on data distribution. Differences between MicroPure-detectable and MicroPure-undetectable MREFs were assessed using the Mann–Whitney U test. Receiver operating characteristic (ROC) curve analysis was performed to evaluate the ability of MREF diameter and depth to discriminate MicroPure detectability. In the ROC analysis, MicroPure-undetectable MREFs were defined as the positive class. The area under the curve (AUC), exploratory classification threshold determined by the maximum Youden index, sensitivity, and specificity were calculated.

Generalized estimating equations (GEE) were used to account for clustering of MREFs within patients. MicroPure detectability was specified as the binary dependent variable, with MREF diameter and depth included as independent variables. An exchangeable correlation structure was applied. Odds ratios (ORs) and 95% confidence intervals (CIs) were reported.

For tibia-level analyses, scatter plots were first examined to assess linear relationships between MREF counts and healing index. Simple linear regression analyses were performed to evaluate the association between early MicroPure-detectable MREF burden and healing index. Because of the limited sample size, multivariable adjustment was not performed and these analyses were considered exploratory. Regression coefficients (β), 95% confidence intervals, and coefficients of determination (R^2^) were calculated. Regression diagnostics, including standardized residual analysis and Cook’s distance, were additionally performed to evaluate potential influential observations. A two-sided p value < 0.05 was considered statistically significant.

## 3 Results

### 3.1 Patient characteristics

A total of 15 patients who underwent tibial bone transport for chronic osteomyelitis under the same orthopedic team and had available paired B-mode and MicroPure ultrasound examinations were included in this study. One patient underwent bilateral treatment, resulting in 16 tibiae available for analysis. The cohort comprised 12 men and 3 women, with a mean age of 44.47 ± 13.89 years (range, 22–65 years). The mean bone defect length was 5.15 ± 2.04 cm (range, 3.00–9.41 cm). The mean external fixation time was 786 ± 381 days, with a median of 635.5 days (IQR, 514.8–1007.5; range, 377–1612 days). A total of 32 ultrasound examinations were performed at 2 and 4 weeks after initiation of distraction. For MREF-level analysis, 1,976 MREFs were evaluated, including 988 MicroPure-detectable and 988 MicroPure-undetectable MREFs. Detailed patient characteristics are presented in Table 1.

**TABLE 1:** Baseline and treatment characteristics of the study cohort.

Variable	Value
Number of patients	15
Age (years)	44.47 ± 13.89
Age range (years)	22–65
Sex (male/female)	12/3
Laterality (unilateral/bilateral)	14/1
Chronic osteomyelitis of the tibia, n	15
Bone defect length (cm)	5.15 ± 2.04
Bone defect length range (cm)	3.00–9.41
Distraction duration (days)	147.6 ± 80.6
External fixation time (days)	786 ± 381; median 635.5 (IQR, 514.8–1007.5)
Number of ultrasound examinations	32
Total MREFs analyzed	1,976
MicroPure-detectable MREFs	988
MicroPure-undetectable MREFs	988

Data are presented as mean ± standard deviation or median (interquartile range) unless otherwise indicated. Patient-level variables were summarized at the patient level, whereas treatment-related variables, including bone defect length and external fixation time, were summarized at the tibia level.

### 3.2 Association of MREF diameter and depth with MicroPure detectability

**FIGURE 2:**
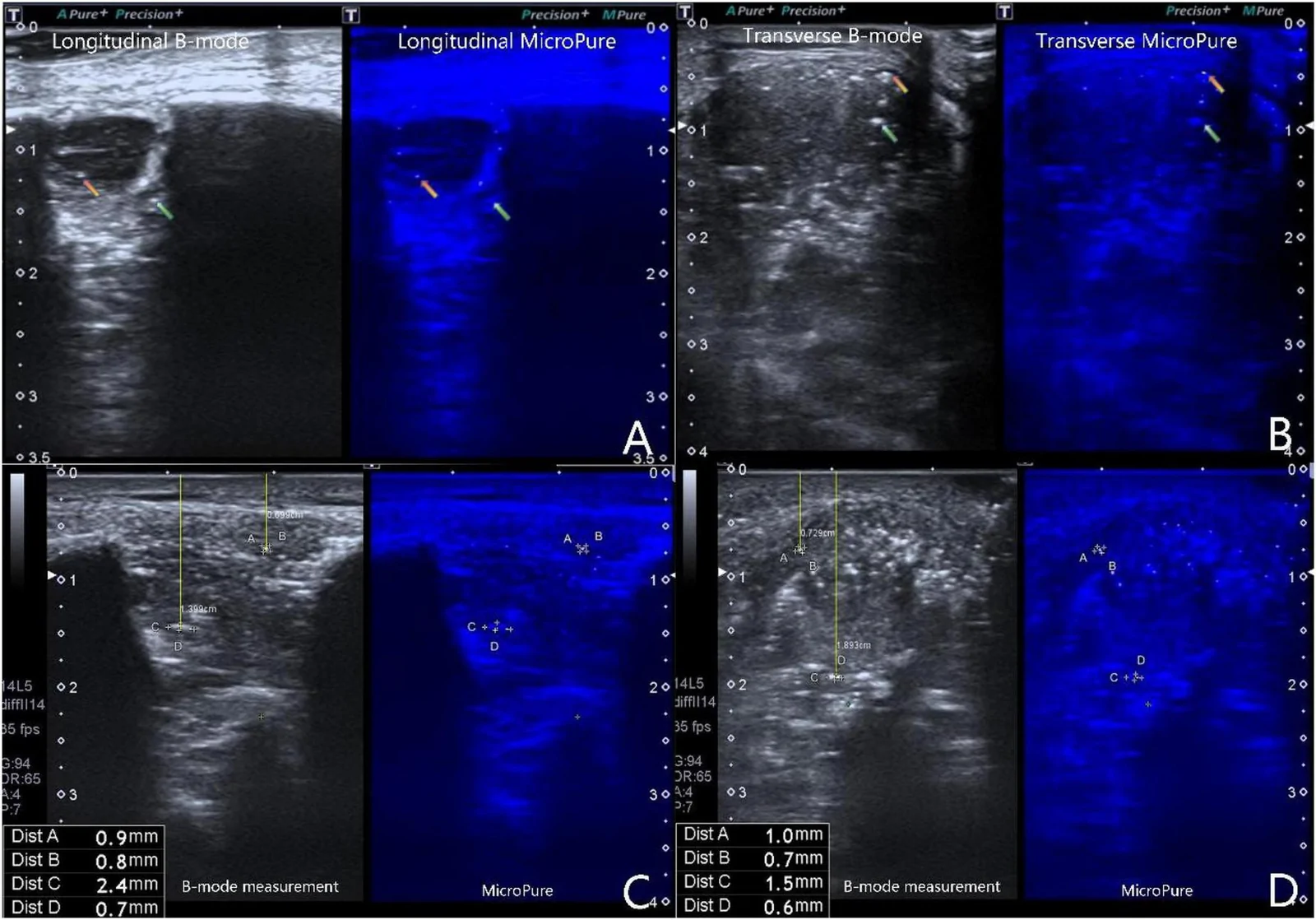
Methodological illustration of MREF classification and measurement in MicroPure ultrasound imaging. **(A,B)** Representative longitudinal and transverse paired B-mode ultrasound and MicroPure images showing a MicroPure-detectable MREF (yellow arrow) and a MicroPure-undetectable MREF (green arrow). **(C)** Representative longitudinal scan and **(D)** representative transverse scan demonstrating measurements of MREF diameter and depth. MREF diameter was measured in both the longitudinal and transverse directions using the ultrasound system calipers, and the larger value was recorded as the maximum diameter (DIST). MREF depth was defined as the vertical distance from the skin surface to the center of the MREF and was measured using the yellow vertical line. The value displayed adjacent to the yellow vertical line indicates the measured MREF depth. These measurements were used for subsequent analyses of factors associated with MicroPure detectability.

**FIGURE 3:**
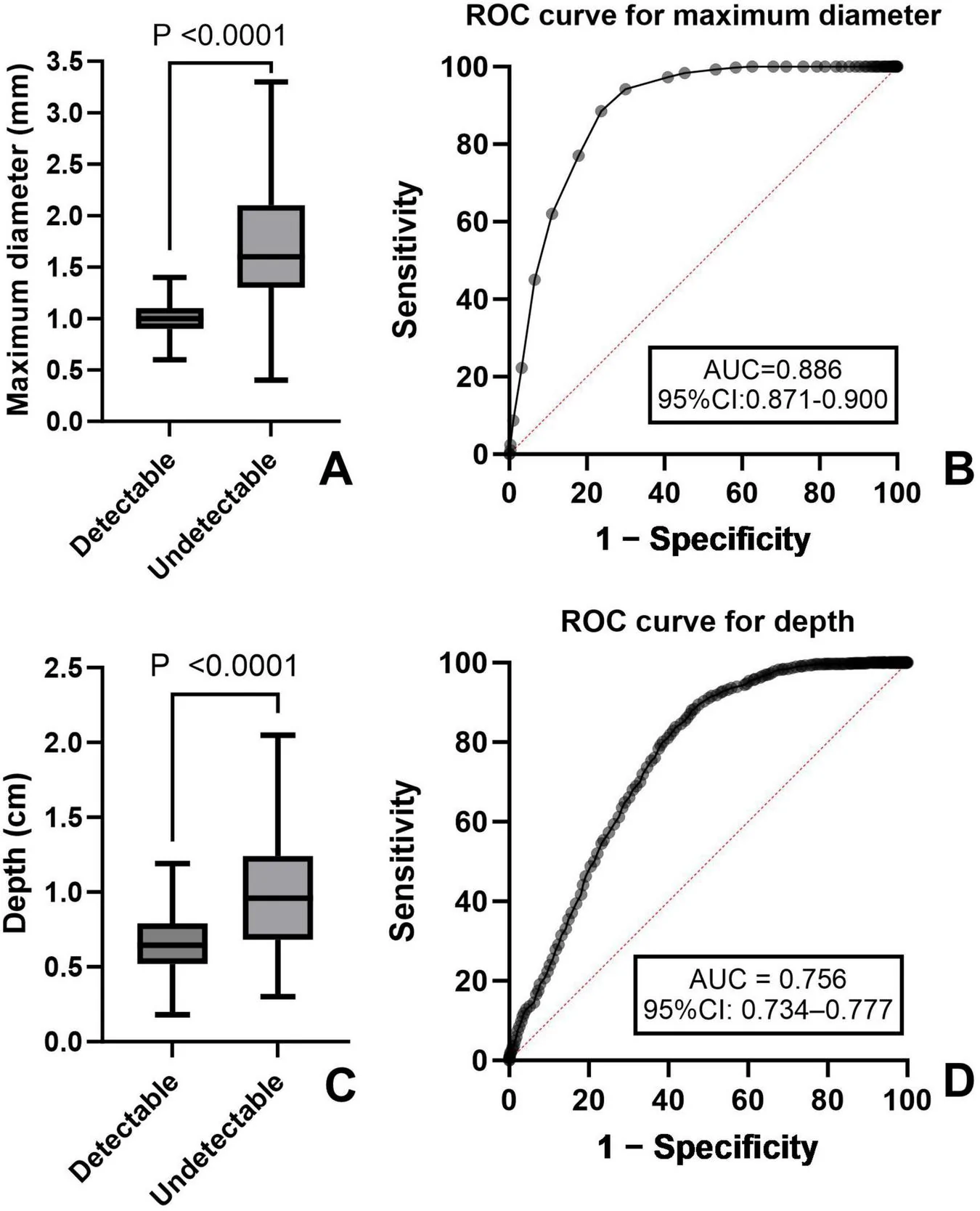
Comparison of MREF diameter and depth between MicroPure-detectable and MicroPure-undetectable MREFs and their discriminatory performance for MicroPure detectability. **(A)** Box-and-whisker plot comparing the maximum diameter of MicroPure-detectable and MicroPure-undetectable MREFs. The MicroPure-detectable group showed significantly smaller maximum diameters than the undetectable group (*P* < 0.001). **(B)** Receiver operating characteristic (ROC) curve of MREF diameter for discriminating MicroPure detectability. The area under the curve (AUC) was 0.886 (95% CI: 0.871–0.900). **(C)** Box-and-whisker plot comparing the depth of MicroPure-detectable and MicroPure-undetectable MREFs. The MicroPure-detectable group showed significantly shallower depths than the undetectable group (*P* < 0.001). **(D)** Receiver operating characteristic (ROC) curve of MREF depth for discriminating MicroPure detectability. The AUC was 0.756 (95% CI: 0.734–0.777). Box plots display the median, interquartile range, and 10th–90th percentiles.

The diameter and depth distributions of MicroPure-detectable and MicroPure-undetectable MREFs are summarized in Table 2 and illustrated in Figures 2, 3. MicroPure-detectable MREFs demonstrated significantly smaller diameters than MicroPure-undetectable MREFs, with median values of 1.0 mm (IQR, 0.9–1.1 mm) and 1.6 mm (IQR, 1.3–2.1 mm), respectively (Z = −29.775, *p* < 0.001). Similarly, the median depth of MicroPure-detectable MREFs was significantly shallower than that of MicroPure-undetectable MREFs [0.645 cm (IQR, 0.52–0.79 cm) vs. 0.96 cm (IQR, 0.68–1.24 cm); Z = −19.672, *p* < 0.001].

**TABLE 2:** Comparison of morphologic characteristics between MicroPure-detectable and MicroPure-undetectable MREFs.

Variable	Detectable (*n* = 988)	Undetectable (*n* = 988)	*Z-*value	*P-*value
Maximum diameter (mm)	1.0 (0.9–1.1)	1.6 (1.3–2.1)	–29.775	< 0.001
Depth (cm)	0.645 (0.52–0.79)	0.960 (0.68–1.24)	–19.672	< 0.001

Data are presented as median (interquartile range). Comparisons between groups were performed using the Mann–Whitney U test.

### 3.3 ROC analysis of exploratory classification thresholds associated with MicroPure detectability

**FIGURE 4:**
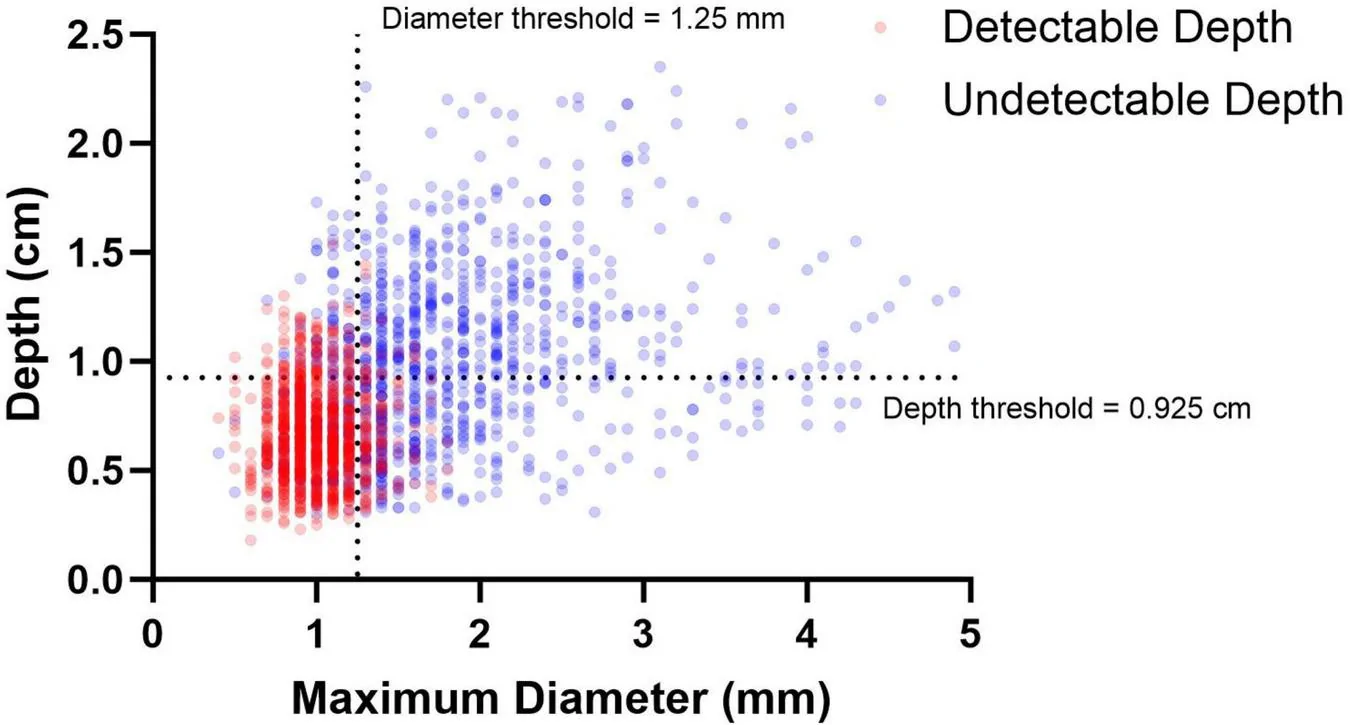
Joint distribution of MREF diameter and depth according to MicroPure detectability. Scatter plot showing the distribution of MicroPure-detectable and MicroPure-undetectable MREFs according to maximum diameter and depth. The vertical dashed line indicates the exploratory diameter classification threshold (1.25 mm), and the horizontal dashed line indicates the exploratory depth classification threshold (0.925 cm) derived from ROC analysis. Most MicroPure-detectable MREFs were concentrated in the lower-left quadrant (diameter < 1.25 mm and depth < 0.925 cm), whereas MicroPure-undetectable MREFs were distributed predominantly outside this region.

Receiver operating characteristic (ROC) curve analyses were performed to evaluate the discriminatory performance of MREF diameter and depth for MicroPure detectability within the sampled B-mode-visible MREFs (Figure 4). In the ROC analysis, MicroPure-undetectable MREFs were defined as the positive class. For MREF diameter, the area under the ROC curve (AUC) was 0.886 (95% CI, 0.871–0.900), indicating good discriminatory performance. The exploratory classification threshold determined by the maximum Youden index was 1.25 mm, with a sensitivity of 76.3% and a specificity of 88.6%. For MREF depth, the AUC was 0.756 (95% CI, 0.734–0.777), indicating moderate discriminatory performance. The exploratory classification threshold was 0.925 cm, corresponding to a sensitivity of 54.1% and a specificity of 88.1%. These thresholds represent the discriminatory characteristics of MicroPure detectability within the sampled B-mode-visible MREFs rather than definitive technical detection limits. Detailed ROC results are presented in Table 3.

**TABLE 3:** Exploratory classification thresholds associated with MREF diameter and depth for MicroPure detectability and cluster-adjusted generalized estimating equation analysis.

Variable	AUC (95% CI)	Exploratory classification threshold	Sensitivity (%)	Specificity (%)	Youden index	OR (95% CI)	*P*-value
Maximum diameter (mm)	0.886 (0.871–0.900)	1.25	76.3	88.6	0.649	0.002 (0.001–0.005)	< 0.001
Depth (cm)	0.756 (0.734–0.777)	0.925	54.1	88.1	0.422	0.089 (0.016–0.492)	0.005

AUC, area under the curve; CI, confidence interval; OR, odds ratio. Receiver operating characteristic analysis was performed to evaluate the discriminatory performance of MREF diameter and depth for MicroPure detectability. MicroPure-undetectable MREFs were defined as the positive class in ROC analysis. Generalized estimating equation analysis included MREF diameter and depth simultaneously while accounting for clustering of MREFs within patients.

### 3.4 Generalized estimating equation analysis of factors associated with MicroPure detectability

Generalized estimating equation (GEE) analysis was performed to evaluate whether MREF diameter and depth were independently associated with MicroPure detectability while accounting for clustering of MREFs within patients. After adjustment for both variables, MREF diameter remained significantly associated with MicroPure detectability (OR = 0.002, 95% CI, 0.001–0.005; *p* < 0.001). Likewise, MREF depth was independently associated with MicroPure detectability (OR = 0.089, 95% CI, 0.016–0.492; *p* = 0.005). The GEE results are summarized in Table 3.

### 3.5 Association between early MicroPure-detectable MREF burden and healing outcomes

**FIGURE 5:**
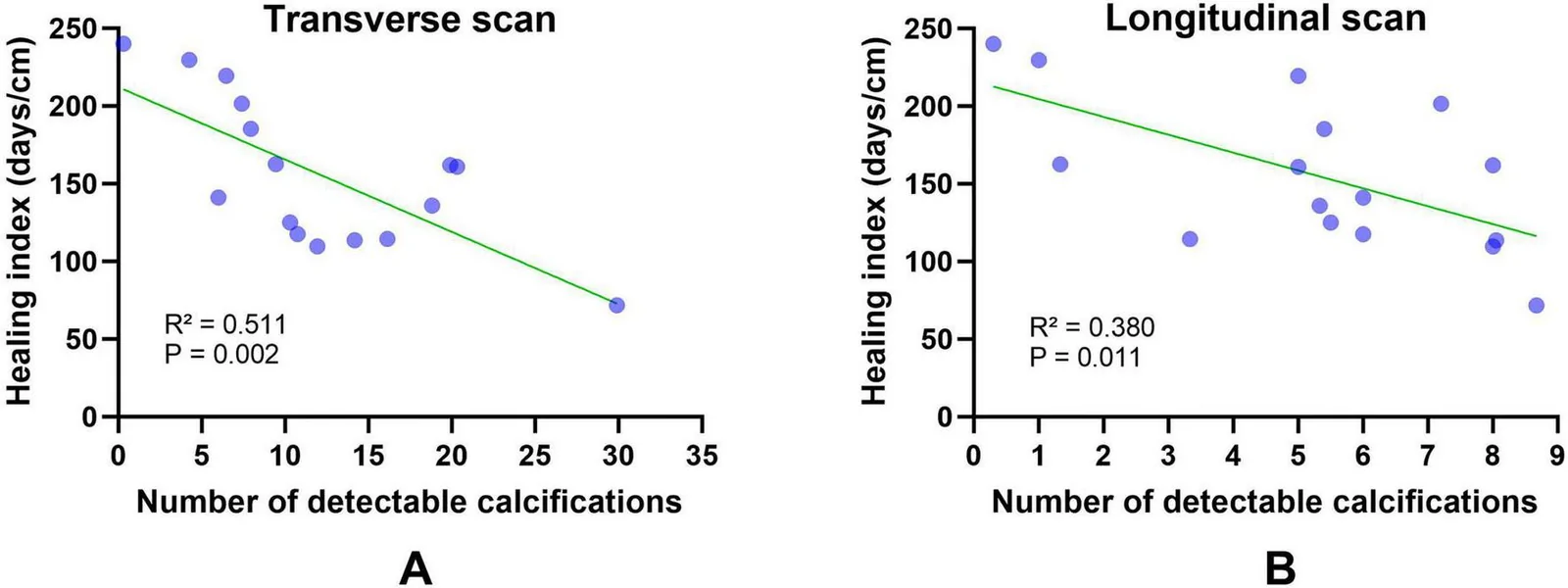
Association between early MicroPure-detectable MREF counts and healing index. **(A)** Linear regression analysis between the number of MicroPure-detectable MREFs on transverse scans and healing index. **(B)** Linear regression analysis between the number of MicroPure-detectable MREFs on longitudinal scans and healing index. A greater number of MicroPure-detectable MREFs was associated with a lower healing index. Because MREF counts were not normalized to scanned tissue volume or area, these findings should be interpreted as exploratory associations between relative MicroPure-detectable echogenic burden and regenerate consolidation.

Tibia-level associations between early MicroPure-detectable MREF counts and healing index are shown in Table 4 and Figure 5. In transverse scans, the number of MicroPure-detectable MREFs demonstrated a significant negative association with healing index (R^2^ = 0.511, B = −4.659, 95% CI, −7.274 to −2.045; *p* = 0.002). Each additional detectable MREF was associated with an estimated difference of approximately 4.66 days/cm in healing index. In longitudinal scans, a significant negative association was also observed (R^2^ = 0.380, B = −11.506, 95% CI, −19.936 to −3.076; *p* = 0.011). Each additional detectable MREF corresponded to an estimated difference of 11.51 days/cm in healing index. Overall, higher early MicroPure-detectable MREF counts were associated with lower healing indices, suggesting a potential relationship between relative MicroPure-detectable echogenic activity and regenerate consolidation. These analyses were considered exploratory due to the limited number of tibiae analyzed. Sensitivity analyses excluding either tibia from the bilateral case yielded consistent associations between early MicroPure-detectable MREF counts and healing index. Regression diagnostic analyses, including standardized residual analysis and Cook’s distance assessment, did not identify influential observations.

**TABLE 4:** Linear regression analyses between early MicroPure-detectable MREF counts and healing index.

Scan orientation	Regression coefficient B (95% CI)	Standardized β	R^2^	*P*-value
Transverse scan	–4.659 (–7.274 to –2.045)	–0.715	0.511	0.002
Longitudinal scan	–11.506 (–19.936 to –3.076)	–0.616	0.380	0.011

The dependent variable was the healing index (external fixation time divided by bone defect length, days/cm). The independent variable was the number of MicroPure-detectable MREFs identified during the early distraction period.

### 3.6 Representative case

**FIGURE 6:**
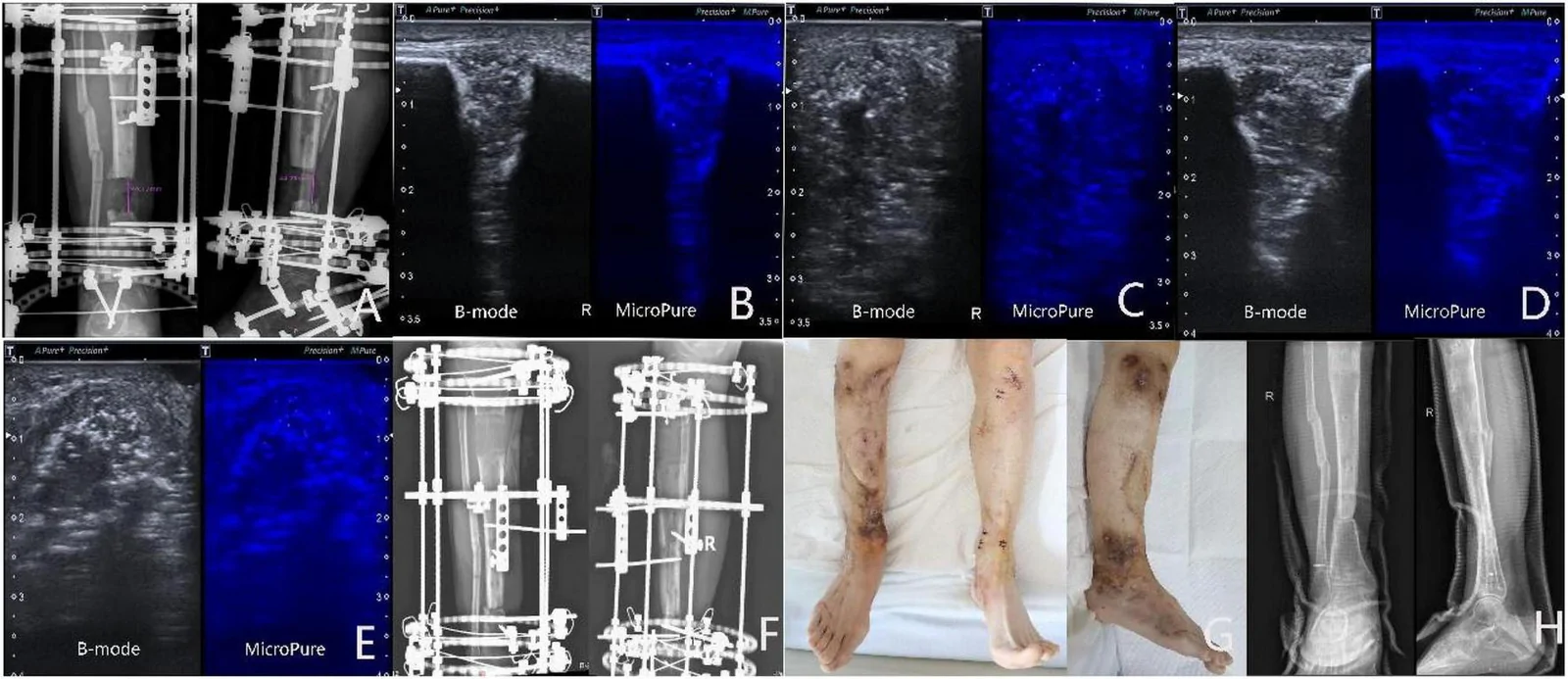
Representative case illustrating MicroPure monitoring during bone transport. **(A)** Postoperative radiograph demonstrating a tibial bone defect of 4.42 cm. **(B,C)** Paired longitudinal and transverse B-mode and MicroPure ultrasound images obtained 2 weeks after distraction, showing multiple MicroPure-detectable MREFs. **(D,E)** Longitudinal and transverse MicroPure images obtained 4 weeks after distraction, demonstrating progressive changes in MicroPure-detectable MREFs within the regenerate bone. **(F)** Radiograph obtained 125 days after surgery showing consolidation of the regenerate bone. **(G)** Clinical photograph after external fixator removal. **(H)** Final radiograph after frame removal demonstrating successful bone regeneration. Written informed consent for publication of the clinical photograph shown in this figure was obtained from the patient. The photograph includes only the affected limb and does not contain any identifiable personal information.

A 31-year-old man with chronic tibial osteomyelitis underwent tibial bone transport for a 4.42-cm tibial defect. MicroPure ultrasound detected six and fourteen MicroPure-detectable MREFs on longitudinal and transverse scans, respectively, at 2 weeks after distraction initiation. At 4 weeks, six MicroPure-detectable MREFs remained visible on longitudinal scans and twelve MicroPure-detectable MREFs were identified on transverse scans. Progressive regenerate consolidation was subsequently confirmed radiographically, with restoration of tibial continuity at 125 days after surgery and successful external fixator removal following complete consolidation (Figure 6).

## 4 Discussion

The present study is, to our knowledge, the first to investigate the performance characteristics of MicroPure ultrasound for detecting MREFs in regenerate bone during tibial bone transport. The principal findings were threefold. First, MicroPure-detectable MREFs were significantly smaller and more superficial than MicroPure-undetectable MREFs. Second, exploratory classification thresholds associated with MicroPure detectability were identified, with thresholds of 1.25 mm for MREF diameter and 0.925 cm for depth. Third, a greater burden of early MicroPure-detectable MREFs was associated with a lower healing index, suggesting a potential relationship between early mineralization-related imaging changes and subsequent regenerate consolidation. A major contribution of the present study is the quantitative characterization of factors associated with MicroPure detectability and the identification of exploratory classification thresholds for interpreting MicroPure findings in distraction osteogenesis. Although MicroPure has been widely used for microcalcification detection in breast and thyroid imaging, its performance characteristics in regenerate bone have not previously been investigated. Our results demonstrated that both MREF diameter and depth significantly influenced detectability. ROC analysis identified exploratory classification thresholds of 1.25 mm for diameter and 0.925 cm for depth, while generalized estimating equation (GEE) analysis demonstrated that both factors remained independently associated with MicroPure detectability. These findings suggest that MicroPure performance in regenerate bone is governed not only by the intrinsic properties of the MREFs themselves but also by the acoustic environment through which ultrasound signals propagate.

From a technical perspective, the observed diameter dependence may be related to the image-processing characteristics of MicroPure. Previous studies have demonstrated that MicroPure enhances the visualization of small calcification-related echoes through dedicated ultrasound image-processing techniques (12, 14, 17). Early mineralization-related echogenic changes observed during distraction osteogenesis may present as small and spatially discrete foci, making them potentially suitable targets for point-enhancement algorithms (18). As these foci enlarge and become more heterogeneous, their imaging characteristics may deviate from the point-target model used by the software, reducing detectability (19, 20).

The influence of depth likely reflects the physical limitations of high-frequency ultrasound imaging (21, 22). Signal attenuation increases with propagation distance, resulting in progressive reductions in signal-to-noise ratio (23, 24). The exploratory depth in this study suggests that greater depth may be associated with reduced MicroPure detectability within the sampled B-mode-visible MREFs. This finding may be particularly relevant in patients with thick soft tissues or complex limb anatomy, where negative MicroPure findings should be interpreted cautiously.

The exploratory classification thresholds identified in this study may provide reference information for clinical interpretation of MicroPure images. First, the absence of a MicroPure signal should not necessarily be interpreted as an absence of mineralization-related imaging changes. MREFs with larger diameters or greater depths may remain visible on conventional B-mode ultrasound while being undetectable on MicroPure imaging. Understanding these technical limitations may help avoid misclassification of regenerate maturation status during follow-up examinations. Second, the exploratory thresholds identified in this study may provide a preliminary framework for standardizing MicroPure assessment in distraction osteogenesis. Rather than relying solely on subjective interpretation of “firefly” signals, clinicians may be able to evaluate findings within the context of known technical performance characteristics. Such standardization could improve the reproducibility of ultrasound-based regenerate assessment across operators and institutions. However, these thresholds were derived and evaluated within the same exploratory cohort and may therefore be subject to optimism bias. Their stability and clinical applicability require validation in independent cohorts.

In addition to characterizing MicroPure detectability characteristics, we explored the relationship between early MicroPure-detectable MREFs and healing outcomes. Both transverse and longitudinal scans demonstrated significant negative associations between early MicroPure-detectable MREF counts and healing index. These findings suggest that regenerate bone demonstrating a greater burden of early MicroPure-detectable MREFs may be associated with more efficient consolidation and shorter external fixation duration. The biological basis for this observation is plausible. During distraction osteogenesis, mineralization represents a critical transition from soft regenerate tissue to mechanically competent bone. MicroPure-detectable MREFs may reflect early mineralization-related imaging changes and may be associated with biological processes involved in regenerate maturation before radiographic mineralization becomes apparent (18). Consequently, the early burden of MicroPure-detectable MREFs may represent a relative imaging indicator of regenerative activity within the distraction gap. From a clinical perspective, patients exhibiting fewer MicroPure-detectable MREFs during the early phase of distraction osteogenesis may represent a subgroup with delayed mineralization-related imaging changes. Such findings may warrant closer surveillance and could potentially support consideration of interventions designed to enhance bone regeneration, including optimization of distraction protocols, low-intensity pulsed ultrasound, or electromagnetic stimulation (25–27). However, whether MicroPure-guided intervention strategies can improve clinical outcomes requires further investigation. Because these associations were observed at the tibia level and were derived from an exploratory analysis without adjustment for potential confounders, the findings should be interpreted cautiously. The healing analysis was performed in a relatively small cohort and should therefore be considered exploratory. Although the observed associations support the potential clinical relevance of MicroPure-detectable MREFs, larger prospective studies will be required to determine whether these findings can be translated into robust prognostic models. Sensitivity analyses excluding either tibia from the bilateral case demonstrated consistent associations between early MicroPure-detectable MREF counts and healing index, suggesting that the observed findings were not primarily driven by the bilateral treatment. In addition, regression diagnostic analyses showed no influential observations, supporting the stability of the exploratory associations.

Current imaging strategies for distraction osteogenesis rely primarily on serial radiography. While radiographs remain indispensable for evaluating alignment and regenerate morphology, their sensitivity for early mineralization is limited (7, 8). More quantitative approaches, including CT-based measurements and dual-energy X-ray absorptiometry, may provide additional information but are associated with radiation exposure, increased costs, and limited practicality for frequent monitoring (28, 29). The findings of this study suggest that MicroPure ultrasound may complement existing imaging modalities by providing a non-invasive method for visualizing early mineralization activity. Because ultrasound is radiation-free, inexpensive, and readily repeatable, MicroPure imaging could potentially be incorporated into routine follow-up protocols during distraction osteogenesis. Beyond bone transport, similar approaches may also be applicable to fracture healing, limb lengthening procedures, and other clinical scenarios requiring early assessment of bone regeneration.

This study has several limitations. First, this was a retrospective single-center study involving a limited number of patients, which may restrict the generalizability of the findings. External validation in larger multicenter cohorts is required. Second, the MREF-level analysis used a balanced sampling strategy to compare MicroPure-detectable and MicroPure-undetectable MREFs. Therefore, the ROC-derived thresholds should be interpreted as exploratory classification values within the sampled B-mode-visible MREFs rather than definitive technical detection limits. Future studies using consecutive sampling strategies, cluster-based internal validation approaches, phantom validation, and external cohorts are required to further evaluate the stability and generalizability of these findings. Third, no histological or micro-computed tomography reference standard was available. Although the detected hyperechoic foci may represent early mineralization-related changes, their biological composition could not be directly confirmed. Other potential sources of echogenic signals, including surrounding soft tissues, fibrosis, gas, or fixation-related interfaces, cannot be completely excluded. Future experimental studies combining MicroPure imaging with micro-computed tomography and histological analyses would help clarify the biological basis of the observed signals. Fourth, depth measurements were derived from exported BMP images using scale calibration methods rather than original DICOM data. Although this approach enabled quantitative analysis, small measurement errors cannot be excluded. Finally, several technical and methodological factors may influence MicroPure detectability and healing outcome interpretation. These include MREF morphology, ultrasound acquisition parameters, patient body habitus, regenerate characteristics, and the absence of normalization of MREF counts according to scanned area or regenerate volume. Therefore, differences observed between transverse and longitudinal scans should be interpreted cautiously, as they may partly reflect acquisition geometry rather than biological differences. In addition, MicroPure classification was determined through joint review by two sonographers rather than independent blinded assessments, and diameter and depth measurements were performed by a single investigator without formal interobserver reliability assessment. Therefore, future studies incorporating standardized imaging protocols, independent image interpretation, multiple-observer measurements, and quantitative normalization strategies are warranted to further validate the reproducibility of MicroPure assessment. Although docking-site management and additional procedures were performed according to clinical requirements, these factors were not specifically analyzed in this exploratory study and may have influenced healing outcomes. Furthermore, potential clinical confounders, including age, comorbidities, smoking status, defect characteristics, and infection severity, were not included in adjusted models because of the limited sample size.

## 5 Conclusion

This study explored the size- and depth-related factors associated with MicroPure detectability of MREFs in regenerate bone during tibial bone transport. Within this cohort, MREF diameter and depth were independently associated with MicroPure detectability, with exploratory classification thresholds of 1.25 mm and 0.925 cm, respectively, within the sampled B-mode-visible MREFs. Furthermore, greater numbers of early MicroPure-detectable MREFs were associated with more favorable healing outcomes. These findings provide quantitative reference values for interpreting MicroPure images and support further investigation of this technique as a non-invasive tool for monitoring mineralization-related changes during distraction osteogenesis.

## Data Availability

The datasets presented in this article are not readily available because the datasets generated and/or analyzed during the current study are not publicly available because they contain potentially identifiable patient information but are available from the corresponding author on reasonable request. Requests to access the datasets should be directed to YZ, yhzhy@139.com.
